# Innovative Use of Waste PET-Derived Additive to Enhance Application Potentials of Recycled Concrete Aggregates in Asphalt Rubber

**DOI:** 10.3390/polym15193893

**Published:** 2023-09-26

**Authors:** Guofu Chen, Yuhao Peng, Nannan Yang, Guohao Xu, Kai Gong, Xiong Xu

**Affiliations:** 1 School of Civil Engineering and Architecture, Wuhan Institute of Technology, Wuhan 430073, China; 22004010051@stu.wit.edu.cn (G.C.); 2001160515@stu.wit.edu.cn (Y.P.); 2104040228@stu.wit.edu.cn (N.Y.); 22204010134@stu.wit.edu.cn (G.X.); 22204010080@stu.wit.edu.cn (K.G.); 2 Key Laboratory of Road Structure and Material of Ministry of Transport (Changsha), Changsha University of Science & Technology, Changsha 410114, China; 3 Hubei Provincial Engineering Research Center for Green Civil Engineering Materials and Structures, Wuhan Institute of Technology, Wuhan 430073, China; 4 School of Civil Engineering, Guang’an Vocational & Technical College, Guangan 638000, China

**Keywords:** waste polyethylene terephthalate, recycled concrete aggregate, asphalt rubber, sustainable asphalt pavement, performance evaluation

## Abstract

Polyethylene terephthalate (PET) drinking bottles, rubber tires, and concrete are the very common municipal solid wastes, which are usually disposed of at landfills and stockpiles and cause continuous damage to the environment. Some studies have indicated that waste PET can be chemically converted into an additive for improving the overall properties of asphalt pavement incorporating natural aggregates, especially the moisture-induced damage resistance. However, it is not clear whether this PET additive still works for asphalt rubber containing recycled concrete aggregates (RCA). To well reveal this issue, this study first adopted a similar way to chemically recycle waste PET into the additive for modifying crumb rubber modified asphalt (CRMA) binder and then mixed the binder with the 13 mm maximum aggregate stone matrix asphalt containing 100% coarse RCA for preparing the mixtures. After a series of physicochemical characterizations of the PET additive, the moisture resistance, rutting resistance, low-temperature cracking resistance, and fatigue resistance of the mixture were systematically evaluated. The results showed that the PET additive is capable of improving the resistance to moisture and high-temperature deformation of asphalt rubber and helps greatly reduce the moisture-induced damage to the interfacial bonding layer. To be more detailed, the residual Marshall stability (RMS) value of RCA-CRMAM/1PET after 72 h of immersion is higher than 85% by contrast to that of RCA-CRMAM (77.1%), while the tensile strength ratio (TSR) value of RCA-CRMAM/1PET shows more than 80% compared to that of 65.2%. In addition, only 1% PET additive can enhance the high-temperature resistance of asphalt rubber containing RCA to rut and allow it to maintain higher resistance to rut after moisture-induced damage. 1% PET additive can help improve the bearing capacity of RCA-CRMAM under a low-temperature environment and delay its fatigue life at small stresses. Generally, with the successful introduction of PET additives to asphalt rubber containing RCA, more durable and sustainable highway pavement can be produced and applied in practice to alleviate the negative impacts caused by waste PET, waste tire rubber, and waste concrete.

## 1. Introduction

Building waste and scrap tires are the typical solid wastes in cities [1,2]. With the advancement of urban modernization, these two kinds of waste are being generated at a fast speed. Landfill will eventually be one of the major options for their disposals, which will definitely lead to a series of environmental problems, such as soil and water pollution [3]. Therefore, the recycling of these wastes has been a hotpot issue all over the world, especially in China.

Previous studies have shown that the concrete waste from buildings can be reprocessed into recycled concrete aggregate for the production of low-class cement concrete [4,5]. In addition, waste tire rubber is also widely used in the construction field [6,7]. For example, Yıldızel et al. [8] mixed waste steel wire (RWSWs) recovered from waste rubber tires into concrete for newly designed reinforced concrete buildings to improve the performance of building components. Zeybek et al. [9] extracted steel fibers from waste tires to prepare fiber-reinforced concrete for improving the mechanical properties of concrete. However, the recovery rate and utilization rate are far lower than the production amount. Thus, it is very important to think about how to efficiently reuse these wastes in different areas under the circular economy.

As a popular paving material, asphalt concrete is widely used in different grades of highway construction due to its high driving comfort and easy-repair characteristics [10,11,12]. For its composition, it mainly includes aggregates and asphalt binder, where the aggregate accounts for more than 90% of the total weight of asphalt concrete [13]. The construction and maintenance of asphalt pavement require a large amount of high-quality natural mineral aggregate [14,15]. However, due to the non-renewable nature of natural mineral aggregate resources, there is a shortage of natural mineral aggregate in many areas [16]. With the research and exploration of road materials by many researchers, it has been found that using recycled aggregate to replace natural mineral aggregate is one of the most effective ways to achieve the sustainable development of asphalt pavement [17,18]. For waste tire rubber, a large number of application practices showed that rubber powder-modified asphalt can improve the high and low-temperature performance of pavement, reduce pavement cracks, and extend the service life of pavement [19,20,21]. Therefore, both kinds of waste can be further applied in the construction of asphalt pavement and solve the problems of environmental pollution and resource shortage.

At present, a lot of work has been performed on the application of RCA in asphalt concrete [22,23]. It is reported that the asphalt concrete prepared by RCA has excellent high-temperature deformation resistance and fatigue performance [24]. However, researchers generally believed that the addition of RCA would reduce the moisture-induced damage resistance of asphalt concrete [25,26]. Gómez-Meijide et al. [27] found that RCA reduces the anti-stripping ability of asphalt mixtures. Mills-Beals et al. [28] found that the mechanical properties of RCA-included asphalt concrete after immersion will be reduced. Hou et al. [29] also confirmed that the moisture resistance of asphalt concrete containing RCA becomes worse with increasing RCA content. Al-Bayati et al. [30] believed that the residual mortar on the RCA surface is the main reason limiting the application of RCA in asphalt pavement. To increase the use of RCA, most of the researchers were and are focusing on the pretreatment of RCA for its quality improvement [31,32]. However, the mentioned pretreatment methods are not convenient and cheap and cannot meet the large-scale paving demand for construction as well. Therefore, it is of great importance to consider an easy method, like mixing additives into the asphalt binder [33], to solve this issue.

PET plastic is the main raw material for manufacturing plastic bottles [34]. With the rapid development of society, PET plastic has become an irreplaceable material in various fields, and its non-degradable waste has also caused serious environmental pollution [35]. In fact, PET plastic has been proven to be a suitable asphalt additive [36], but it is difficult to get practical application in asphalt pavement due to its high melting point (>200 °C) [37]. Previous studies have shown that PET additive, obtained by the aminolysis of waste PET plastic, is a degraded polymeric material with a lower melting point and workability and can be well mixed with asphalt binder, showing a very strong ability to enhance the moisture-induced damage resistance of asphalt mixtures containing natural aggregates [38]. However, there is a research gap to understand if this additive still works on asphalt mixtures containing RCA.

**Figure 1:**
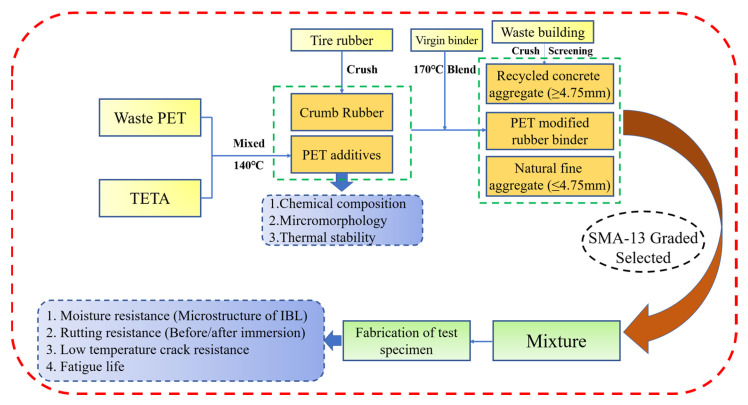
Research flowchart for this study.

To fill this research gap, this study includes the following: waste rubber tires are processed into crumb rubber and mixed with virgin bitumen to prepare rubberized binder; waste PET plastics are aminated to be used as modifiers for rubber asphalt; and coarse aggregates are replaced with RCA in the gradation to produce asphalt concrete. Afterwards, the microscopic morphology, chemical components, and thermal stability of PET additives will be characterized, and then the performance properties of target asphalt mixtures will also be verified in combination with the microstructural analysis of PET additives. To briefly describe the experimental procedures, they are summarized in the flowchart depicted in Figure 1.

## 2. Materials and Methodology

### 2.1. Raw Materials

#### 2.1.1. Asphalt Binder and Aggregates

The asphalt binder is virgin bitumen with a Pen. 70 grade, provided by a local supplier. After testing, the penetration at 25 °C, ductility at 10 °C, and softening point were measured at 69 dmm, 16.6 cm, and 48.2 °C, respectively.

With regard to aggregates, two types of aggregates were used, namely coarse RCA (4.75–16 mm) and fine NA (0–4.75 mm). To better understand their physical properties, the test results of some major indexes are listed in Table 1.

#### 2.1.2. Crumb Rubber

**Figure 2:**
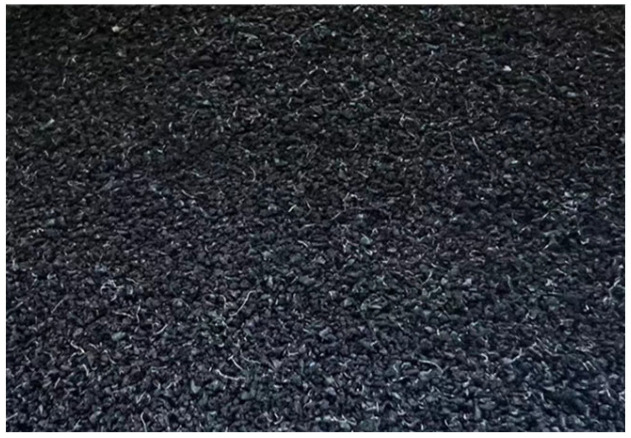
The appearance of crumb rubber.

Crumb rubber (CR) was sourced from the mixed waste car-and-truck tire rubbers, which were provided from a local tire-recycled factory. The CR particles used were the ones obtained from the sieving process with particle sizes between 30 mesh and 50 mesh, as shown in Figure 2.

#### 2.1.3. Waste PET

**Figure 3:**
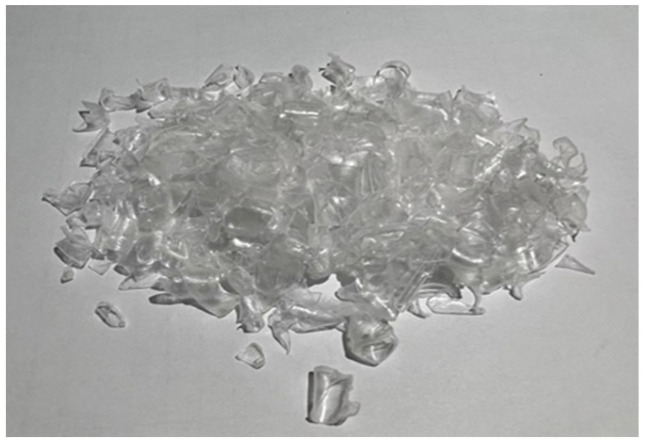
The appearance of waste PET flakes.

Waste PET was purchased from a local plastic-recycled supplier and obtained from different drinking bottles. Before use, these waste PET bottles were mechanically crushed and ground into some small pieces with a maximum size of 10 mm after removing the labels and caps, as displayed in Figure 3.

### 2.2. Preparation of PET Additive

**Figure 4:**
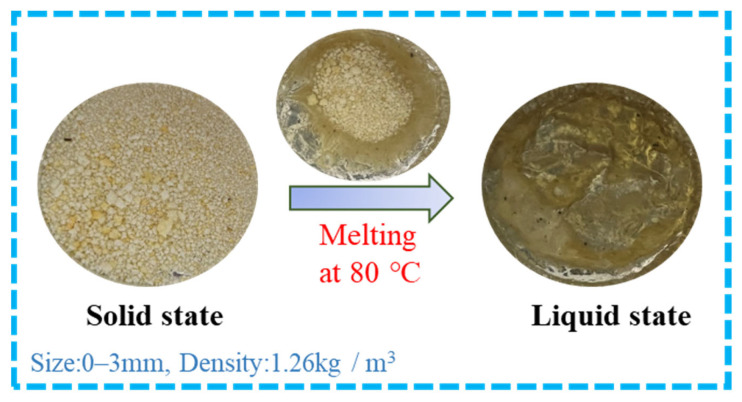
Physical state changes of PET additive at 80 °C with minutes.

Following similar procedures in our previous studies [39,40], waste PET pieces were chemically treated using triethylenetetramine (TETA) at 140 °C for 2 h in the plant. Afterwards, the collected degradation products were repeatedly washed and then dried in a 35–45 °C oven. Finally, the dried products were ground into fine powders as additives for use. To preliminarily understand the changes in physical state, the PET additive was thermally disposed at 80 °C for visual observations (Figure 4). It is clear that the physical state develops at stages like the following: (a) solid state before thermal treatment; (b) solid-liquid state within seconds; and (c) liquid state within minutes.

### 2.3. Preparation of PET Additive-Modified Rubberized Asphalt

First of all, 18% CR, by weight of virgin binder, was added into and mixed with the molten virgin asphalt binder at 170 °C for 30 min with a shearing rate of 2000 rpm to prepare CR modified asphalt (CRMA) binder. Afterwards, 1% and 3% PET additives, by total weight of CRMA binder, were blended into the above mixture for 5 min to prepare the respective binders (CRMA/1PET and CRMA/3PET). At last, their primary property results are presented in Table 2.

### 2.4. Preparation of RCA-CRMAM/PET Mixtures Incorporated with RCA

**Figure 5:**
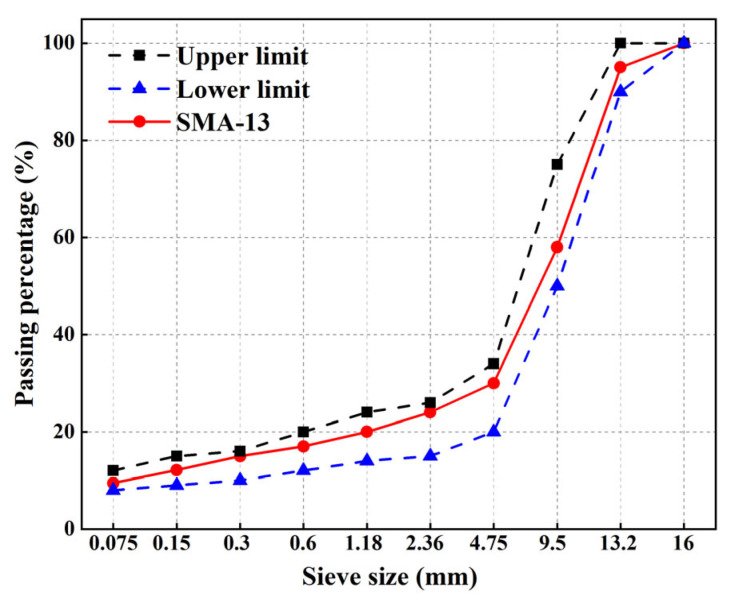
SMA-13 gradation curve for the job mix of target asphalt rubber.

The aggregate degradation of asphalt rubber is often the gap-graded type, which serves to provide spaces for the thermal expansion of crumb rubber, guaranteeing the mixture volume stability after finishing the paving works. Stone matrix asphalt (SMA) degradation is one of the gap-degraded types that has been widely applied in the preparation of asphalt rubber. Therefore, as small additions of PET additive show less impacts on rubberized binder in terms of physical properties, including viscosity, the SMA-13 degradation was selected for the mix design of asphalt rubber to prepare target mixtures. The aggregate gradation for SMA-13 was used as shown in Figure 5. According to the mix design, the optimum asphalt content for the SMA-13 mixture was 6.9%. The mixtures were named RCA-CRMAM, RCA-CRMAM/1PET, and RCA-CRMAM/3PET, respectively, based on different contents of PET additive (0, 1%, and 3%).

### 2.5. Comprehensive Characterizations of PET Additive

To overall evaluate the physicochemical properties of the PET additive, the following tests were adopted: (a) microscopic morphology; surface appearances of waste PET before and after treatment were detected and analyzed by the field-emission scanning electron microscope (ZEISS Gemini SEM 300, Oberkochen, Germany) under vacuum conditions; (b) the molecular structure was characterized by FTIR spectra using the Nicolet Impact 420 FTIR spectrometer (Waltham, MA, USA); (c) the thermal behavior was analyzed by a thermogravimetric analyzer (Netzsch STA 449, Hanau, Germany), and the sample was heated from ambient temperature to 800 °C at a rate of 10 C/min; and (d) the crystalline structure was characterized by X-ray diffraction patterns (from 10° to 80°, Cu-Kαradiation, λ = 1.54 Å) using a D8-Advance powder X-ray diffractometer operating at 40 kV and 30 mA, with a scanning rate of 6°/min and a scanning step of 0.02 (XRD, Bruker AXS, Karlsruhe, Germany).

### 2.6. Performance Tests of Asphalt Mixtures

#### 2.6.1. Moisture-Induced Damage Resistance

Moisture-induced damage is one of the key factors that cause the deterioration of asphalt mixture performance. The invasion of water reduces the cohesion between asphalt binder and RCA and increases the probability of stripping between recycled aggregate and binder. The residual Marshall stability tests (RMS tests) and freeze-thaw split tests (F-T tests) were used to evaluate the moisture-induced damage resistance of RCA-CRMAM. Both test methods involved making six Marshall-samples with a diameter of 101.6 mm and a height of 63.5 mm, where the air void of RMS samples and F-T samples were set to be 4~4.5% and 7~8%, respectively. Subsequently, the residual Marshall stability (RMS) and tensile strength ratio (TSR) were calculated to quantify the moisture-induced damage resistance of the asphalt mixture.

According to ASTM D6927, the RMS samples were immersed in a water bath at 60 °C for 0.5 h, 24 h, 48 h, and 72 h, respectively. The residual Marshall stabilities (RMS) of target samples are obtained following Equation (1):

(1) $$RMS=\frac{{MS}_{i}}{{MS}_{0}}\times 100\%$$

where *MS*_0_ (kN) is the Marshall stability of target samples after soaking at 60 °C for 0.5 h, and *MS_i_* (kN) is the Marshall stability of target samples after soaking at 60 °C for *i* hours (i = 24, 48, and 72).

In accordance with AASHTO T283, the target samples were subject to standard freeze-thaw damage for i cycles (freeze at −18 °C for 16 h and thaw at 60 °C for 24 h as a cycle). The indirect tensile strength (ITS) of each specimen was tested, and the indicator TSR was determined according to Equation (2):

(2) $$TSR=\frac{{ITS}_{i}}{{ITS}_{0}}\times 100\%$$

where ${ITS}_{0}$ (MPa) is the ITS of asphalt mixtures before the freeze-thaw cycles, and ${ITS}_{i}$ (MPa) is the ITS of asphalt mixtures after freeze-thaw cycles (i = 1, 2, and 3).

#### 2.6.2. Wheel Tracking Test

A wheel tracking test is commonly used to evaluate the permanent deformation of asphalt pavement at evaluated temperatures. To well understand the resistance of asphalt pavement to moisture damage during the wheel tracking process, this study first fabricated the test slabs sized at 300 mm × 300 mm × 50 mm and then put those slabs into the water bath at 60 °C for 1 d. Afterwards, the slabs were taken out to the 60 °C test chamber, where they stayed for 4 h. Finally, the test was carried out under the following conditions: wheel pressure of 0.7 MPa, rolling speed of 42 passes/min, and rolling time of 60 min. The dynamic stability (DS) and the residual DS (RDS) are obtained following Equations (3) and (4):

(3) $$DS=\frac{15N}{d_{2}-d_{1}}$$

(4) $$RDS=\frac{{DS}_{\mathrm{wet}}}{{DS}_{\mathrm{dry}}}\times 100\%$$

where DS refers to the dynamic stability, pass/mm, *d*_1_ and *d*_2_ refer to the respective rutting depth of asphalt mixtures at 45 min and 60 min, mm, and RDS is the residual DS percentage of asphalt mixtures after immersion, %.

#### 2.6.3. Low-Temperature Crack Resistance

The long-temperature property of asphalt mixtures is generally characterized using a trabecular bending test at −10 °C. This study first prepared the target specimens with a size of 250 mm × 35 mm × 30 mm, and according to JTG E20/T 0715, these prepared specimens were moved to the −10 °C test chamber of the UTM-100 testing machine and kept for 4 h. Afterwards, the test was carried out to contact the middle part of the specimen with a loading rate of 50 mm/min until fracture failure. The evaluation parameters, namely low-temperature flexural-tensile strength (*R_B_*), flexural-tensile strain (*ε_B_*), and blending stiffness modulus (*S_B_*), are calculated following Equations (5)–(7).

(5) $$R_{B}=\frac{3LP_{B}}{2bh^{2}}$$

(6) $$\varepsilon_{B}=\frac{6hd}{L^{2}}$$

(7) $$S_{B}=\frac{R_{B}}{\varepsilon_{B}}$$

where *R_B_* is the flexural tensile strength, MPa; ε_B_ is the maximum flexural tensile strain, *uε*; *S_B_* is the bending stiffness modulus, MPa; *b* is the width of the specimen, mm; h is the height of the specimen, mm; *L* is the span of the specimen, mm; P_B_ is the peak load, N; and *d* is the deflection when the specimen was broken, mm.

#### 2.6.4. Fatigue Resistance

**Figure 6:**
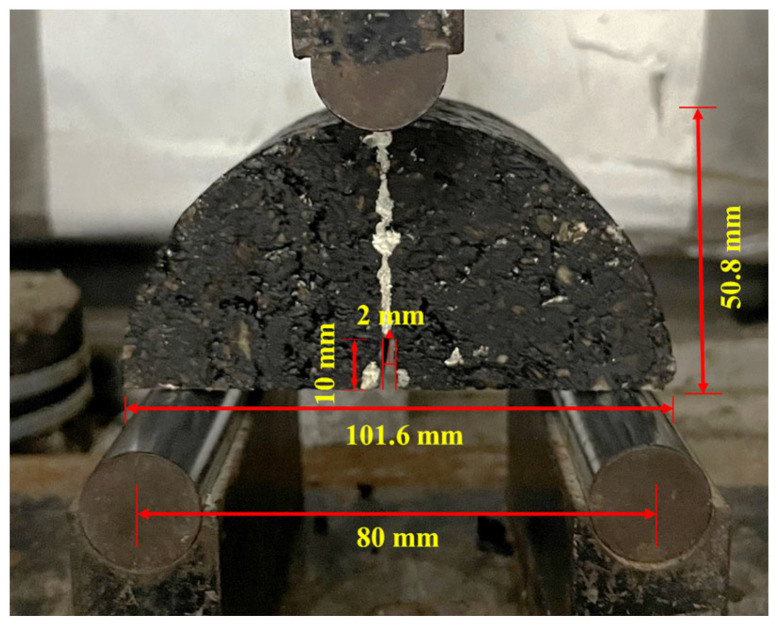
Specimen size and loading process.

Fatigue resistance is commonly used to assess the durability of asphalt pavement. In this study, the Marshall specimens were first prepared and cut in half for the selected repeated semicircular bending test (R-SCB) [41]. Prior to the test, the peak load of the target asphalt mixtures was determined. During the test (see Figure 6), the measurement setups were as follows: the test temperature was 25 °C, the hold time was 5 h, and the applied loads were 0.3, 0.4, 0.5, 0.6, and 0.7 times of the critical applied load. The relevant indexes are calculated following Equations (8)–(10).

(8) $$\sigma_{0}=\frac{P_{c}}{2rt}$$

(9) $$K_{IC}=Y_{I}\sigma_{0}\sqrt{\pi a}$$

(10) $$N_{f}=A\times K_{IC}^{-n}$$

where $\sigma_{0}$ is the applied stress, MPa; $P_{c}$ is the critical applied load, *N*; *r* is the radius, m; *t* is the thickness, m; $K_{IC}$ is the critical stress intensity factor in mode *I*, MPa × m^0.5^; $Y_{I}$ is the normalized stress intensity factor in mode I, MPa × m^0.5^; a is the creak length, m; $N_{f}$ is the fatigue life, cycle; *A* is the dimensionless coefficient to reflect the resistance to fatigue; and n is the dimensionless coefficient to reflect the fatigue susceptibility.

### 2.7. Field emission Scanning Electron Microscope (FESEM)

**Figure 7:**
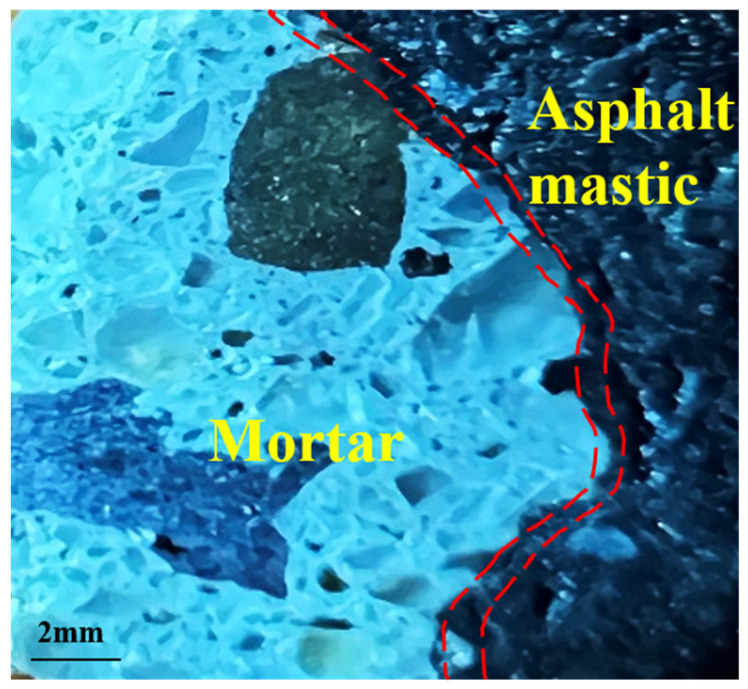
Microscopic structure of the asphalt specimen.

In order to observe the state of the Interface Bonding Layer (IBL) between RCAs after moisture-induced damage, a hand-held cutting machine was used to cut from the Marshall specimen after moisture-induced damage, and later, a cube sample with a side length of 10 mm was taken. The sample contains a complete observation area of RCA-Asphalt Binder-RCA (see Figure 7). Then the sandpaper was used to polish the observation surface until it was smooth, and then the surface was cleaned with anhydrous ethanol. After drying, the observation samples were obtained.

## 3. Results and Discussion

### 3.1. Physicochemical Properties of the PET Additive

**Figure 8:**
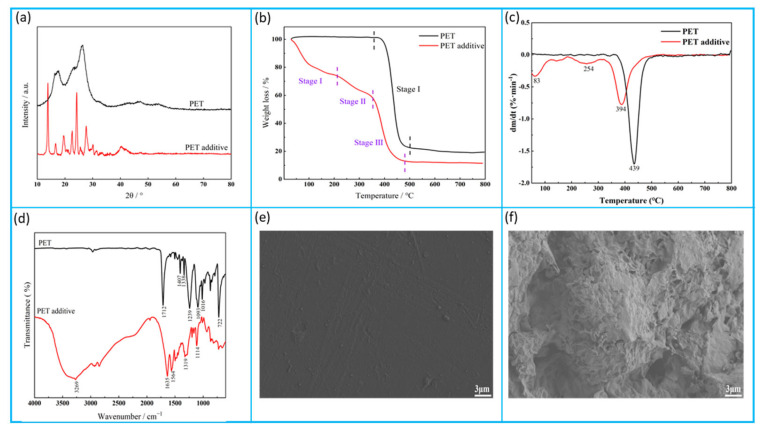
Overall physicochemical property evaluation of the PET additive: (**a**) XRD patterns; (**b**,**c**) TG/DTG analysis; (**d**) FTIR analysis; and (**e**,**f**) SEM images.

Figure 8 displays an overall physicochemical property evaluation of the PET additive. From the XRD results shown in Figure 8a, it is found that PET exhibits broad diffraction peaks at 2θ = 16.2°, 17.2°, 24.6°, and 26.4°, and by contrast, PET additive has narrower and higher diffraction peaks at 2θ = 13.8°, 16.6°, 19.5°, 22.6°, 24.3°, and 27.7°, corresponding to crystal plane indexes at (110), (−211), (220), (012), (−203), and (211), respectively. This result indicated that the PET additive is a monoclinic crystal system that is totally different from the triclinic crystal system of PET plastic.

Furthermore, from the TG and DTG results (see Figure 8b,c), after being degraded for waste PET, the initial decomposition temperature drops from around 400 °C to 83 °C, and the mass loss increases from one to three. On this basis, the mass loss duration of PET additive is divided into three stages: (a) the first stage at 50~200 °C, presenting the mass loss of approximately 23%, mainly from free water and crystal water; (b) the second stage at 200~360 °C, showing the mass loss of around 14%, mainly from the degradation products of waste PET through ammonolysis; and (c) the third stage at 360~470 °C, exhibiting the mass loss up to around 46%, mainly from the decomposition of higher molecular weight oligomers formed by the ammonolysis of waste PET [42].

Figure 8d presents the difference between PET and PET additives in molecular structure. It can be found that, by contrast, the characteristic peak of 1712 cm^−1^ in the infrared curve of PET plastic disappears, the characteristic peak of C–O–C at 1000~1300 cm^−1^ decreases, and the characteristic peak at 722 cm^−1^ decreases. In addition, the new peaks at 3269 cm^−1^ and 1635 cm^−1^ attributed to N–H stretching and bending vibrations in amino groups, the absorption peak belonged to N–H bending vibration in the amide group at 1564 cm^−1^, as well as the peaks related to C–N stretching vibration at 1319 cm^−1^ and C–H stretching vibration at 2936 cm^−1^ and 2849 cm^−1^ in amine, are clearly observed in the infrared curve of PET additive. The above phenomena indicate that PET plastic gets successfully degraded into the new products with an amine-based structure, promoting the improvement of the lipophilic property of the PET additive towards asphalt binder and cohesion between binder and RCA aggregates [43]. In addition, the amide group in the PET additive can form a hydrogen bond with the hydroxyl group on the surface of RCA, which is conducive to enhancing the adhesion between asphalt and RCA.

Figure 8e,f exhibit the SEM images for comparing the waste PET before and after treatment. It is obvious that after chemical treatment of waste PET, the smooth surface turns into a rough surface with several pores. This observation indirectly proves that PET additives have a larger specific surface area, which can be easily blended with asphalt binder.

### 3.2. Moisture Damage Resistance

**Figure 9:**
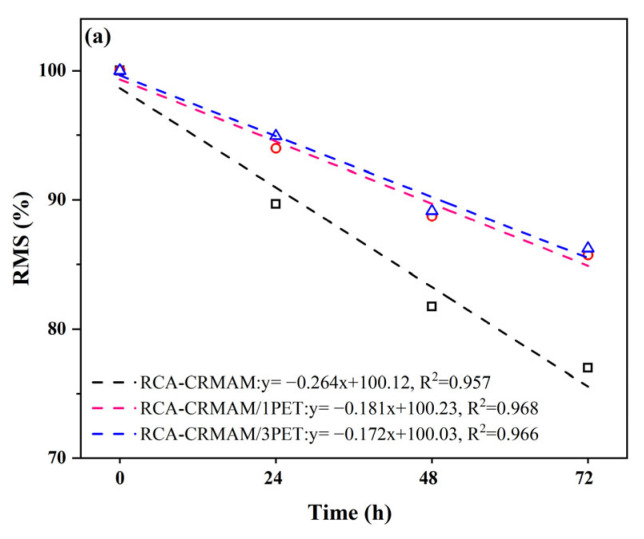

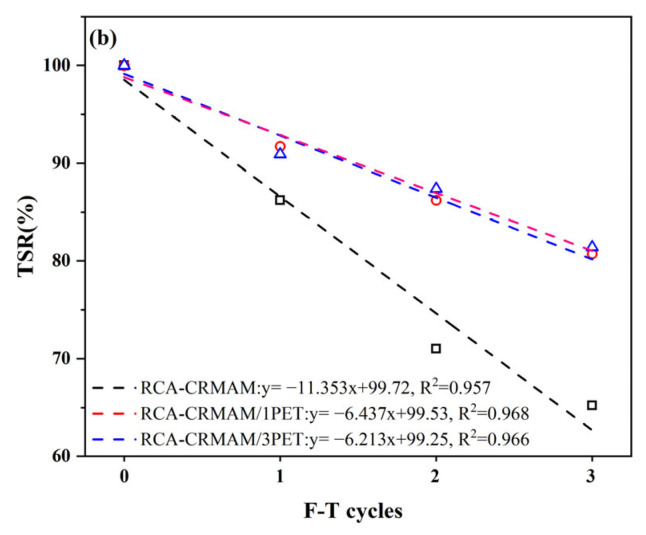
Moisture resistance of RCA-CRMAM containing PET additives (**a**) RMS and (**b**) TSR.

Figure 9 presents the results of the hot water immersion test and freeze-thaw test. As shown in Figure 9a, with the extension of immersion time, the RMS values of all specimens decrease to varying degrees. After 48 h of immersion, the RMS values of RCA-CRMAM/1PET and RCA-CRMAM/3PET are 8.5% and 9.0% higher than those of RCA-CRMAM. After the immersion time is extended to 72 h, the RMS value of RCA-CRMAM goes to 77.1%, while the RMS values of RCA-CRMAM/1PET and RCA-CRMAM/3PET are presented as higher than 85%. From the slope of the fitting straight line, the RMS decline rate of RCA-CRMAM is nearly 50% higher than that of RCA-CRMAM/1PET and RCA-CRMAM/3PET.

As reflected in Figure 9b, it shows that the trend of strength ratio in the freeze-thaw splitting test is similar to that of residual moisture resistance assessment. After one F-T cycle, the TSR values of RCA-CRMAM/1PET and RCA-CRMAM/3PET were 6.3% and 5.4% higher than those of RCA-CRMAM, respectively. After three F-T cycles, the TSR values of RCA-CRMAM are 65.2%, while the TSR values of RCA-CRMAM/1PET and RCA-CRMAM/3PET are more than 80%. Similarly, the slope of the fitted line shows that the decline rate of the TSR value of RCA-CRMAM is almost twice as high as that of RCA-CRMAM/PET. Furthermore, a similar result, given by Sanchez-Cotte et al. [23], stated that the TSR value of asphalt mixtures containing more than 15% RCA cannot satisfy the technical threshold value of 80% after one F-T cycle, which, by contrast, indicated the technical method in this study has significant merits in largely recycling RCA into asphalt pavement with more superior moisture-induced damage resistance.

The comprehensive test results of immersion damage and freeze-thaw damage stated that PET additives can significantly improve the moisture resistance of RCA-CRMA, especially under harsh freeze-thaw damage modes where increasing content is not conducive to working effectively [44]. As far as all the results are concerned, this is because the introduction of PET additive brings its special amino structure into rubber asphalt to enhance intermolecular forces, including van der Waals force, enhance the adhesion between asphalt binder and an RCA weak old mortar surface, and effectively reduce the risk of water erosion in the interface transition zone between CRMA and RCA.

### 3.3. FESEM

**Figure 10:**
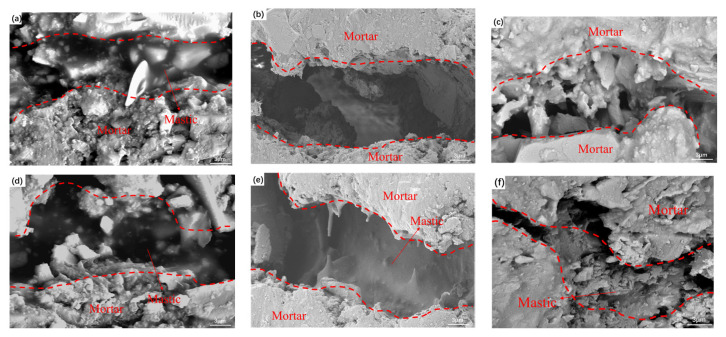
FESEM images of RCA-CRMAM/1PET (**d**–**f**) and RCA-CRMAM (**a**–**c**) (**a**,**d**) before moisture damage, (**b**,**e**) after 48 h immersion damage, and (**c**,**f**) after 3 F-T damage.

In order to further understand the enhancement mechanism of the PET additive on the moisture-induced damage resistance of RCA-CRMAM, Figure 10 illustrates the damaged morphology of the interface bonding layer after hot water damage and F-T damage. It can be seen from Figure 10a,d that the asphalt rubber provides good bonding performance between RCA particles before moisture damage. Compared with hot water damage, freeze-thaw damage will not only lead to the stripping of asphalt rubber, but also increase the width of the tack coat (see Figure 10b,c). The addition of PET additives effectively alleviates this phenomenon (see Figure 10e,f). These microstructure images demonstrate that PET additive can improve the adhesion between RCA and greatly reduce the stripping of asphalt rubber caused by water, which is due to the improved adhesion of asphalt binder after PET additive modification. This also verifies the test results in Section 3.2.

### 3.4. Rutting Resistance

**Figure 11:**
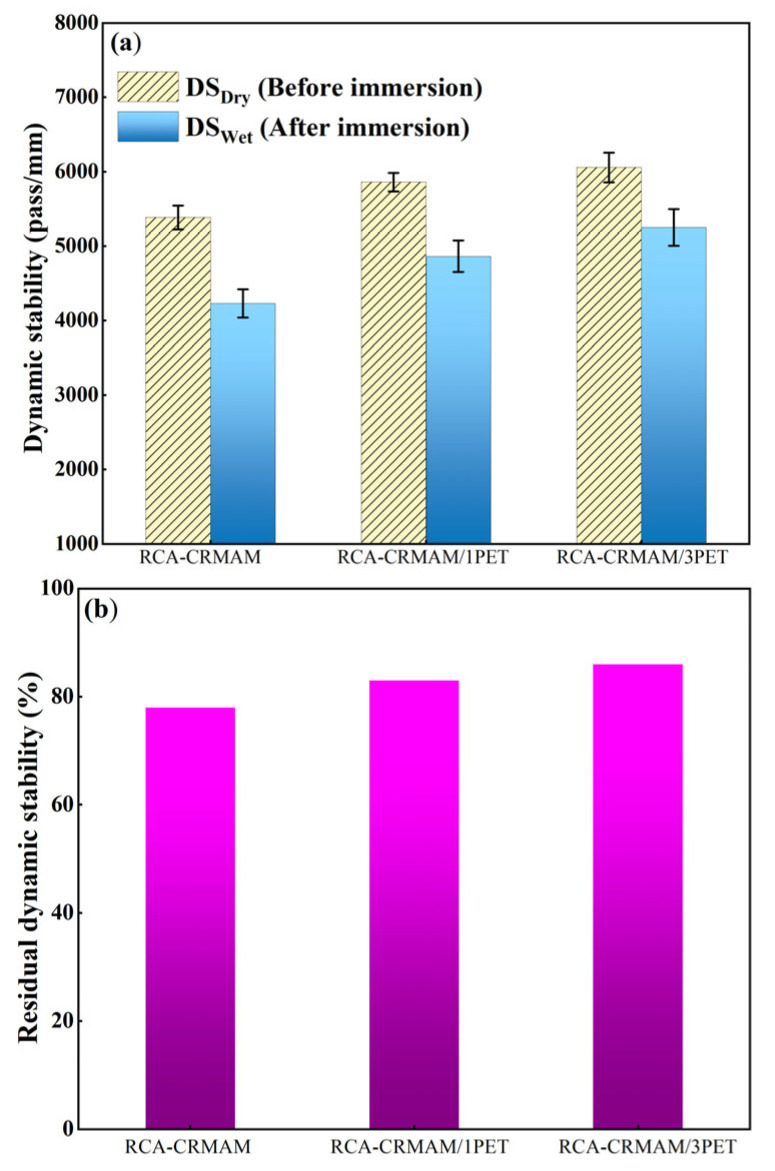
Effects of water immersion on DS (**a**) and RDS (**b**) of RCA-CRMAM containing PET additive.

Figure 11 shows the DS and RDS of the mixture before and after immersion. From Figure 9a, it is clear that the DS values of the three samples decrease to varying degrees after immersion treatment, among which, as the dosage of PET additive is 1% and 3%, the DS value of RCA-CRMAM increases from 4228 pass/mm to 4864 pass/mm and 5250 pass/mm, respectively. In addition, Figure 9b shows that the RDS values of RCA-CRMAM/1PET and RCA-CRRMAM/3PET are 83% and 86%, showing 6.4% and 10.3% higher than RCA-CRMAM, respectively. These results indicate that only 1% of PET additive can enhance the high-temperature resistance of asphalt rubber containing RCA to rut and allow it to maintain higher resistance to rut after moisture-induced damage.

**Figure 12:**
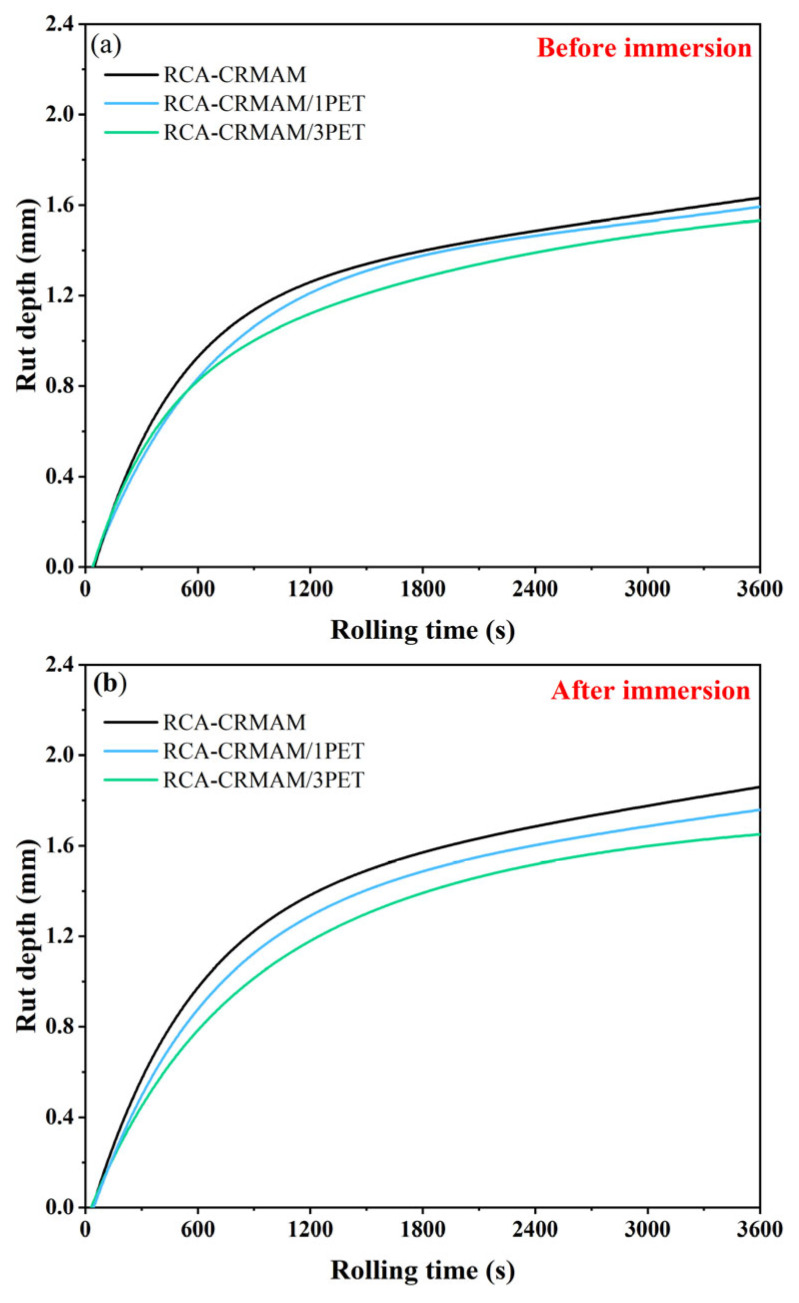
Rut depth development of RCA-CRMAM containing PET additive (**a**) before immersion and (**b**) after immersion.

In order to further understand the wheel tracking test results, the development of ruts with rolling time before and after immersion is shown in Figure 12. It can be found that the rut depths of RCA-CRMAM, RCA-CRMAM/1PET, and RCA-CRMAM/3PET are 1.635 mm, 1.595 mm, and 1.545 mm, respectively, and the rut development speed of RCA-CRMAM is slightly faster than that of RCA-CRMAM/PET. Figure 12b shows that the rut depth and development speed have increased in varying degrees after immersion. The final rut depth of RCA-CRMAM is increased to 1.866 mm, while the rut depths of RCA-CRMAM/1PET and RCA-CRMAM/3PET are 6.5% and 9.5% less than RCA-CRMAM, respectively. These phenomena demonstrated that 1% PET additive can effectively inhibit the development of rut, and 3% PET additive can further reduce the rut depth a little bit and has very little effect on the speed of rutting development. The reason is that PET additive improves the softening point and adhesion of rubberized binder [45], improves the deformation resistance and adhesion of asphalt rubber to RCA, and reduces the sensitivity of RCA-CRMAM to water, which in excess can only improve the deformation resistance of RCA-CRMAM.

In comparison with an existing study, Mills-Beale and You [28] showed that the resistance of asphalt mixtures to rut becomes worse as the RCA content increases from 0% to 75%, for which the rut depth of an asphalt mixture containing 75% RCA after 4000 cycles of loading comes to close to 6-mm rut depth. But, by contrast, this study presented that the rut depths of asphalt rubber containing 100% coarse RCA and 1% PET additive are both less than 2.0 mm before and after immersion, which indicated PET additive helps maintain the high-temperature resistance of asphalt mixtures in dry and wet environments.

### 3.5. Low-Temperature Crack Resistance

**Figure 13:**
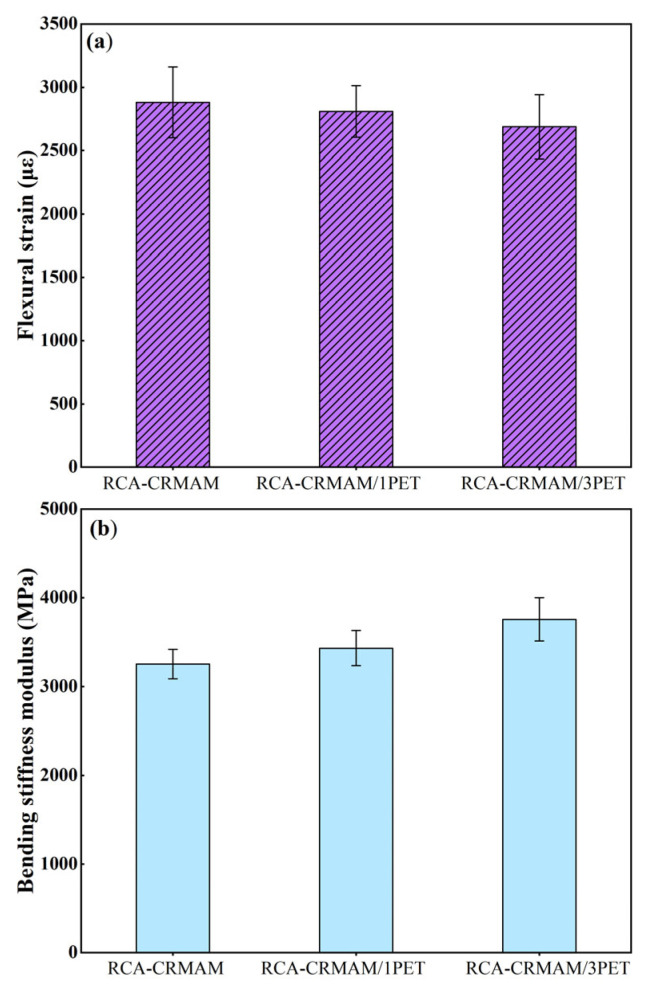
Low-temperature cracking resistance performance of RCA-CRMAM containing PET additive. (**a**) Flexural strain; (**b**) bending stiffness modulus.

Figure 13 shows the low-temperature crack resistance test results of the specimens. It is clear that the maximum tensile strain of the three specimens is higher than 2500με. The addition of the PET additive reduced the maximum tensile strain of RCA-CMARM. The bending tensile strain of RCA-CMAM/3PET and RCA-CMARM/1PET is 6.7% and 2.5% lower than that of RCA-CMAM, respectively. It can also be obtained that the stiffness modulus of the specimen is improved by the incorporation of PET additives, and the stiffness modulus of RCA-CMARM/3PET is 9.4% and 15.4% higher than that of RCA-CRMAM/1PET and RCA-CRMAM, respectively. These results show that the PET additive slightly reduced the low-temperature deformation capacity of RCA-CRMAM, but it helped improve the bearing capacity of RCA-CRMAM under a low-temperature environment. The reason is that the addition of PET additive enhances the adhesion between rubber asphalt and RCA, making the mixture have excellent bending resistance.

**Figure 14:**
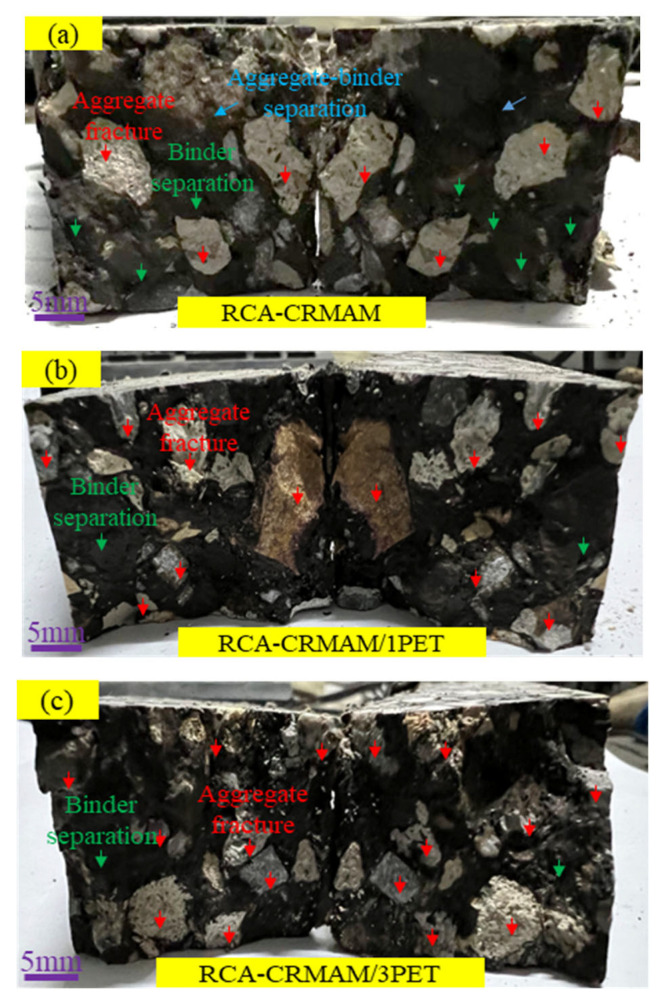
Macroscopic fracture morphology of each mixture at a low temperature: (**a**–**c**) are RCA-CRMAM, RCA-CRMAM/1PET and RCA-CRMAM/3PET, respectively.

Figure 14 presents the fracture morphology for RCA-CRMAM with and without PET additives. The RCA-CRMAM fracture image (see Figure 14a) shows the rubberized binder leaves on the surface of RCA aggregates; very few fractured areas of rubberized binder and RCA aggregates can be observed. With the addition of 1% PET additive (see Figure 14b), the fractured area of RCA aggregates is significantly increased, and the binder fractured areas are found somewhere. Turning to Figure 14c, the 3% PET additive still has further improvement on the interface fracture of RCA-CRMAM. This is because the rubberized binder will become hard and brittle at low temperatures, and the fracture behavior will be that as the adhesive strength of the binder is poor, the binder will be broken and detached from the surface of RCA aggregate, and as the adhesive strength of the binder is strong, the RCA aggregates will get damaged prior to the CRMA binder. This demonstrates that the addition of PET additives can effectively improve the adhesive strength of CRMA binder and aggregates.

### 3.6. Fatigue Life

**Figure 15:**
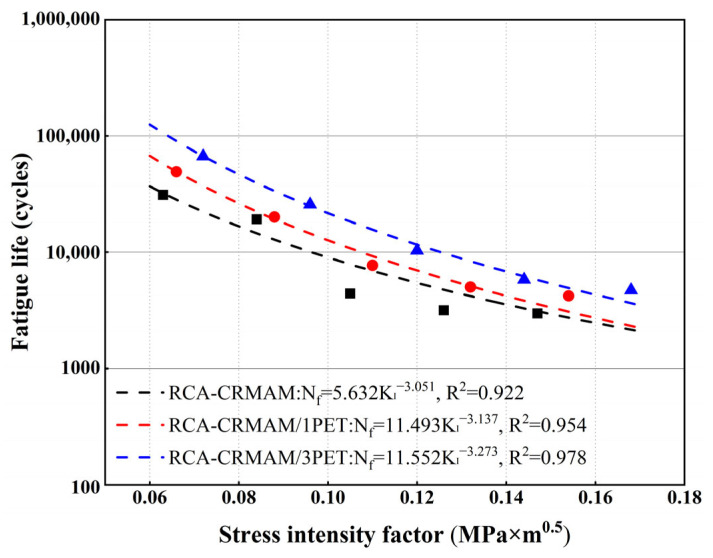
Fatigue life of RCA-CRMAM containing PET additives.

Figure 15 displays the relationship between fatigue life and the stress intensity factor of specimens at 25 °C. The points in the figure represent the number of loading cycles of the specimen under the corresponding stress intensity factor. It is believed that the fatigue life of the mixture gets greatly improved after the addition of PET additives, especially in the low stress zone (such as 0.06~0.10 MPa × m^0.5^), and with the increasing stress, the differences between RCA-CRMAM/PET and RCA-CRMAM become less significant. Meanwhile, it can be found that for RCA-CRMAM, the A value, given from the fitted equations of fatigue life, increases significantly from 5.632 to 11.493 and 11.552, respectively, after 1% and 3% PET additives are added. Correspondingly, the n values remain close to each other, showing a slight change from 3.051 to 3.137 and 3.273, respectively, as 1% and 3% PET additives are added to the rubberized binder. The results show that the fatigue life of RCA-CRMAM can be significantly prolonged by adding PET additive, especially under small stress, and more additions of PET additive will significantly delay the fatigue life of RCA-CRMAM. This is due to the fact that after PET additive is blended with CRMA, the amino and amide groups in its molecular structure promote the physical and chemical interactions between CRMA and RCA, which helps enhance the adhesion of CRMA binder to RCA, but it slightly restricts the deformation capacity of CRMA binder to cause the mixture to be more sensitive to load changes.

## 4. Conclusions

This study first adopted the aminolysis method to chemically recycle waste PET into the additive for modifying the rubberized asphalt binder and then mixed the binder with the graded aggregates containing 100% coarse RCA for preparing the mixtures. After a series of physicochemical characterizations of the PET additive, the moisture resistance, rutting resistance, low-temperature cracking resistance, and fatigue resistance of the mixture were systematically evaluated. The main conclusions are as follows:

- Physicochemical property results indicate that PET additive is the substance with a monoclinic crystal structure, which has not only three different components with an initial mass loss temperature of 83 °C but also amine-based molecular structures and rough surfaces with several pores, potentially contributing to better improving the adhesion of modified binder to RCA aggregates in comparison with waste PET plastic.
- Moisture-induced damage results stated that regardless of water immersion or freeze-thaw conditions, PET additives can greatly improve the moisture-induced damage resistance of RCA-CRMAM because of the strengthened interface bonding layers. Only 1% PET additive can promote RCA-CRMAM to meet the specification requirements after suffering from 72 h water immersion and three freeze-thaw cycles.
- Wheel track test results suggest that only 1% PET additive can enhance the high-temperature resistance of asphalt rubber containing RCA to rut and allow it to maintain higher resistance to rut after moisture-induced damage. One percent PET additive can effectively inhibit the development of rut, and 3% PET additive can further reduce the rut depth a little bit and has very little effect on the speed of rutting development.
- Low-temperature property results demonstrate that PET additive can slightly reduce the low-temperature deformation of RCA-CRMAM, but correspondingly, it can help improve its bearing capacity.
- Fatigue life results reflected that PET additives contribute to delaying the fatigue life of RCA-CRMAM, especially under small stress, and their excessive use will not work significantly for further extension of fatigue life.

Overall, this study suggests using waste PET-derived additives to better recycle RCA in asphalt pavement on a large scale, as it brings about some merits in engineering properties, particularly moisture-induced damage resistance. The research findings demonstrate only 1% PET additive can be performed well to improve the moisture-induced damage resistance of asphalt rubber containing RCA. If it is considered in practice, the engineers should take its dosage control into account.

## Figures and Tables

**Table 1:** Physical properties of aggregates.

Item	Coarse RCA	Fine NA	Requirements	Specification
	4.75–16 mm	0–4.75 mm		
Apparent density (kg/m^3^)	2.54	2.74	≥2.50	ASTM C127
Water absorption (%)	6.9	-	≤3	ASTM C127
Crushing value (%)	20.4	-	≤28	ASTM C942
Los Angeles abrasion loss (%)	26.4	-	≤35	ASTM C131
Fine aggregate angularity (%)	-	52.4	≥30	AASHTOTP33
Sand equivalent (%)	-	70.3	≥60	ASTM D2419

**Table 2:** Primary properties of rubberized asphalt binders with different PET additive dosages.

Type	Ductility at 5 °C, cm	Penetration at 25 °C, dmm	Softening Point, °C
CRMA	5.5	48	58.6
CRMA/1PET	4.2	41	58.9
CRMA/3PET	3.8	32	59.4

## Data Availability

The data presented in this study are available on request from the corresponding author.
